# Optimization of Hybrid Sol-Gel Coating for Dropwise Condensation of Pure Steam

**DOI:** 10.3390/ma13040878

**Published:** 2020-02-15

**Authors:** Riccardo Parin, Michele Rigon, Stefano Bortolin, Alessandro Martucci, Davide Del Col

**Affiliations:** Department of Industrial Engineering, University of Padova, Via Venezia 1, 35131 Padova, Italy; riccardo.parin@phd.unipd.it (R.P.); michele.rigon.4@phd.unipd.it (M.R.); stefano.bortolin@unipd.it (S.B.); davide.delcol@unipd.it (D.D.C.)

**Keywords:** dropwise condensation, hydrophobic surfaces, sol–gel film, aluminum substrate

## Abstract

We developed hybrid organic–inorganic sol–gel silica coatings with good durability in harsh environment (high temperatures, high vapor velocities) and with slightly hydrophobic behavior, sufficient to promote dropwise condensation (DWC) of pure steam. DWC is a very promising mechanism in new trends of thermal management and power generation systems to enhance the heat transfer during condensation as compared to film-wise condensation (FWC). The sol–gel coatings have been prepared from methyl triethoxy silane (MTES) and tetraethyl-orthosilicate (TEOS) and deposited on an aluminum substrate. The coatings were optimized in terms of precursor ratio and annealing temperature highlighting potentials and limits of such mixtures. A comprehensive surface characterization before and after saturated steam condensation tests has been performed and related to the thermal measurements for evaluating the heat transfer augmentation as compared to FWC obtained on untreated aluminum surfaces. The results showed that the developed hybrid organic-inorganic sol–gel silica coatings are promising DWC promoters.

## 1. Introduction

In the heat exchange industry, metals are the most widely used materials because of their thermomechanical characteristics, such as sturdiness, high strength to weight ratios and thermal conductivity. From copper to steel, through aluminum, these kinds of materials are pretty resistant against vapor corrosion; on the contrary, they are highly susceptible to corrosion in aggressive environments. In marine applications, for instance, the mutual effect of oxygen, magnesium, calcium, high level of chloride, combined with the presence of organic compounds, pollutants and biological activity creates very hostile conditions. In these environment corrosion mechanism must be deeply understood to guarantee the metal’s strength [1]. In order to extend the possible applications of metals and protect them against corrosion, a wide range of coatings have been applied in the past. Among them, hybrid organic-inorganic sol–gel silica coatings seem to be the most promising ones due to high versatility, simple processability, and the possibility to tailor their characteristics, ranging from those typical of polymers, like flexibility and softness, to those typical of ceramics, like hardness, abrasion resistance, and brittleness [2]. Sol–gel processing temperature is low, frequently close to room temperature, allowing to avoid thermal volatilization and/or degradation of entrapped species, e.g., organic inhibitors or organic functionalization [3]. Since liquid precursors are used, it is possible to deposit the coatings on bare substrates with complex or exotic shapes, with a wide range of thickness, and without the need for machining or melting [4]. Furthermore, sol–gel films are usually synthesized through “green” technology approaches, using reagents that do not introduce impurities into the final product, do not produce hazardous side-products, and, usually, avoiding chemical routes that involve dangerous goods, such as fluorinated molecules. The introduction of organic groups yields another interesting characteristic to sol–gel coatings: control of the wettability, which is a matter of great interest in the thermal industry. Controlling the wettability of surfaces leads to an increase in the thermal performances during condensation, allowing us to achieve dropwise condensation (DWC) instead of film-wise condensation (FWC). During FWC a thick continuous layer of condensate wets the surface, resulting in a large thermal resistance that must be overcome, whereas this layer is substituted by a droplet population in the DWC mode [5,6]. The increase of heat transfer coefficient (HTC) is between 6 and 10 times compared to FWC [7]. If the HTC increases, high conductivity material like copper can be substituted with less noble materials like aluminum and steel without affecting the global heat transfer efficiency. The low cost, lightweight, high thermal and electrical conductivity of aluminum gives it a remarkable industrial and economic importance. Several studies regard the application of sol–gel coating to aluminum substrates in order to protect them in corrosive environments. Many of its applications are practicable due to its natural tendency to form a passivating aluminum oxide layer, which can also be artificially generated by anodizing the substrate. However, this passivating layer deteriorates in aggressive media, such as chloride, which results in pitting corrosion. A broad range of research is being actively pursued to develop environmentally benign organic coatings for corrosion protection such as hybrid sol–gel coating.

Vapor or water at high temperature (>100 °C) etches the aluminum forming boehmite structures [8,9] enhancing the wettability of the surface, thus efforts must be addressed to protect the surface. Several studies are related to the production of superhydrophobic surfaces using sol–gel coating [10,11,12,13,14,15,16], very limited studies rely on the quasi-hydrophobic surface [7], and, furthermore, those treatments are not tested in harsh environment, e.g., high temperatures and high vapor velocity. Both quasi-hydrophobic and superhydrophobic surfaces allow the DWC mode, but research on superhydrophobic ones is very far from obtaining satisfactory results in terms of surface treatment life [17]. The durability of the coatings deposited on aluminum surface is also an open field of research, very few authors were able to achieve satisfactory results [18,19], still not sufficient for the scale-up to industrial processes. The present paper starts from the sol–gel coatings developed by Innocenzi et al. [20] where tetraethyl orthosilicate (TEOS) and methyl triethoxy silane (MTES) were utilized as silica precursors, aiming at extending the promising results obtained by Parin et al. [7]. Different reagent concentrations and baking temperatures were analyzed in order to understand the limits and the advantages of the sol–gel MTES/TEOS hybrid coatings for the promotion of DWC.

## 2. Materials and Methods

### 2.1. Materials

Methyl triethoxy silane (MTES, 99%), tetraethyl orthosilicate (TEOS, 98%) and ethanol (puriss. p.a., absolute, ≥99.8%) were supplied by Sigma Aldrich (Schnelldorf, Germany). Hydrochloric acid solution (1N) and bidistilled water were purchased from Labochimica (Padova, Italy). All the chemicals were used as received without any further purification. Substrates were high purity aluminum (AW 1050, minimum aluminum content 99.50%), 50 mm high and 20 mm wide, polished on the condensation side before coating’s deposition, following a standard metallographic technique. The lapping step is performed using abrasive SiC papers P500, P800, P1200, and P4000 grit and then polishing step is done with clothes and diamond suspension of 6 μm and 1 μm, in order to produce a mirror-like surface. Polishing allows us to obtain a smooth surface, with standardized roughness, and to remove all native oxides and dirty eventually deposited during the sample’s manipulation. A complete description of the mirror-like bare Aluminum surface’s features is reported in [7].

### 2.2. Coating Preparation

Sol–gel silica solutions were synthesized in acidic conditions. Ethanol, silica precursors, water and hydrochloric acid were added in a beaker under vigorous stirring in a molar ratio of EtOH/TEOS + MTES = 2, H_2_O/TEOS + MTES = 4, HCl/TEOS + MTES = 0.01. After 30 min of stirring, the sol is diluted with EtOH to get a final SiO_2_ nominal concertation of 1.3 mol/L and deposited by dip-coating onto the bare aluminum substrate with a withdraw speed of 4.8 cm/min. Then, the samples were heat-treated in the air inside a furnace at 200 °C, 300 °C or 400 °C for 1 h, with 5 °C/min heating ramp. Coatings with different compositions were analyzed and listed in Table 1.

According to the literature [20], the composition of each film was labeled with alphanumeric code: M_x_T_y_,_number, where x is the molar fraction of MTES, y is the molar fraction of TEOS, and the number corresponding to the baking temperature of the sample.

### 2.3. Coating Characterizations

Samples properties were evaluated by different techniques. Wettability with and without the coating was evaluated using the sessile drop technique [21] with an in-house goniometer composed of a DCC1545M CMOS sensor camera (Thorlabs GmbH^®^, Bergkirchen, Germany) coupled with a MVL7000 zoom lens (Thorlabs GmbH^®^, Bergkirchen, Germany). Contact angles (CA) were collected measuring both the angles during the advancing phase (θ_a_) and the receding phase (θ_r_) of a 5μL water droplet. The measurement was carried out on different positions for each sample and the average angle was adopted as reported value. The chemistry of the surface was investigated by Fourier-Transform Infrared (FTIR) spectroscopy measurements in ATR mode, performed in the 550–4500 cm^−1^ range using a Jasco FT-IR 4200 with Jasco ATR PRO410-S (Jasco Europe S.R.L., Cremella, Italy) attachment. Thickness was measured by ellipsometry technique using a J.A. Woollam V-VASE Spectroscopic Ellipsometer (J.A. Woollam, Lincoln, Nebraska, USA) at three different angles of incidence (55°, 65°, 75°) in the 300–1700 nm wavelength range. The fitting of the experimental data (Ψ and Δ functions) was done using WVASE^®^ ellipsometry data software (version 3.717, J.A. Woollam, Lincoln, NE, USA), implementing a “Cauchy Transparent Dispersion” model over the Aluminum’s substrate model. Finally, a scanning electron microscope was used to observe the features of the surface.

### 2.4. Condensation Tests

The samples were placed in a thermosiphon flow loop developed for the study of steam condensation on vertical metallic substrates. The experimental apparatus and the data reduction technique were fully described in [16,21], hence only some fundamental details are reported here. The condensation tests were performed in steady-state in order to evaluate the duration of treatments; all the treatments sustained DWC for different timing, after it, the condensation mode changes to FWC indicating the degradation of the coating. The test was considered ended when part of the surface was flooded (FWC) since the transition is irreversible. Then, the treated surface was maintained in a steady environment for the whole condensation test, typical thermodynamic conditions were 105 °C saturation temperature (1.20 bar saturation pressure) and vapor speed equal to 2.6 m s^−1^. The back of the sample was cooled by water coming from a thermal bath, instead. Both the coolant mass flow rate and the coolant inlet temperature were maintained constant during the condensation test, allowing to impose a desired heat flux to the surfaces. Typical value of the heat flux was 400 kW m^−2^. The heat transfer performances were measured by calibrated thermocouples placed within the sample. Each aluminum sample has six T-type thermocouples arranged in pairs along the direction of the steam: two at the inlet, two in the middle and two at the outlet of the specimen. Each pair has a sensor located at a distance of *z_1_* = 1.3 mm and the other one at *z_2_* = 2.8 mm depth from the condensing surface. A sketch of the experimental rig and of the tested samples is provided in the Supporting Materials (Figure S1). The heat flux was measured locally by applying the Fourier law to the thermocouples.

(1) $$q_{loc}\;=\;\lambda_{al}\frac{\Delta T}{\Delta z},$$

where $\lambda_{al}$. is the aluminum thermal conductivity. The temperature difference Δ*T* was measured between the thermocouple placed at 1.3 mm and the one placed at 2.8 mm from the condensing wall, where Δz. is the distance between the two thermocouples. The local heat transfer coefficient (HTC) was evaluated as:

(2) $$HTC\;=\;\frac{q}{\left(T_{sat}\;-\;T_{wall}\right)},$$

where *T_sat_* is the saturation temperature and *T_wall_* is the wall temperature, extrapolated from the thermocouple’s readings [16]. Each parameter was reported as the mean of 480 readings recorded at 1 Hz and with bars whose length represents the expanded combined uncertainty, determined according to the ISO Guide to the Expression of Uncertainty in Measurement [22] (coverage factor *k* = 2).

## 3. Results and Discussion

### 3.1. Layers Characterization

#### 3.1.1. Evaluation of Wettability Proprieties

**Figure 1:**
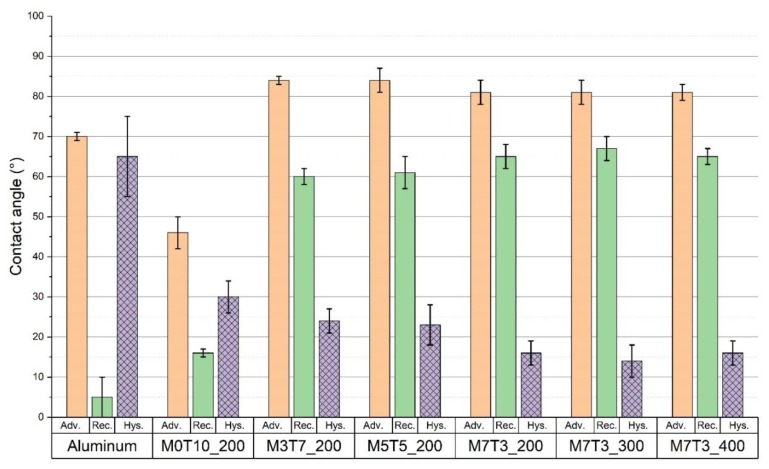
Contact angles of the bare aluminum and the aluminum coated with silica film with different methyl triethoxy silane (MTES)/tetraethyl orthosilicate (TEOS) ratios and different thermal annealing before condensation tests.

**Figure 2:**
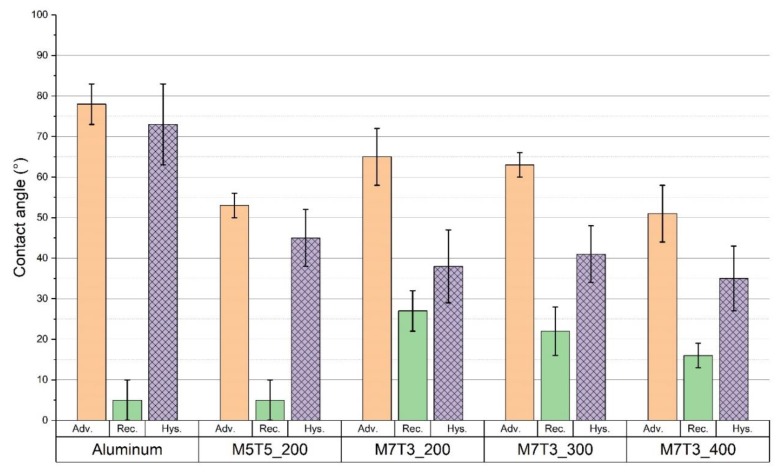
Contact angles of the bare aluminum and the aluminum coated with silica film with different MTES/TEOS ratios and different thermal annealing after condensation tests.

Contact angles of bare and coated aluminum are reported in Figure 1 (before the condensation test) and in Figure 2 (after the condensation test). All the angles were calculated as the average of a minimum of five measurements on the same type of film. The bare aluminum surface shows a hydrophilic behavior, characterized by the formation of a flattened drop in the receding phase, undeterminable with the sessile drop method (see also Table S2). Due to the introduction of organic groups in the sol–gel silica skeleton, wettability gradually decreases, because of the increasing of the receding contact angle, and turning the hydrophilic behavior of silicon oxide closer to hydrophobicity. As a result, hysteresis decreases (see patterned purple columns in Figure 1), helping the drops to slide off/roll off easer from the surface.

From Figure 1, a slight gradual decrease of the hysteresis angle is detected increasing the MTES content, while clear change in wettability due to different baking temperatures, and so different degrees of reticulation of the coating (see Section 3.1.2), is not identifiable. Bearing in mind this trend, surfaces with only silica coating or with silica modified with a lower content of organic modifiers were considered not suitable for DWC mode and they were not tested for this specific application.

After running the condensation tests the coating appears seriously compromised with the exposition of the metal surface and formation of corrosion phenomena. In these conditions, the hysteresis of the contact angle abruptly increase (see Figure 2), and the samples were not able anymore to support the DWC mode.

An example of the effect of vapor condensation on the treatment is reported in Figures S3 and S4. The as-deposited coating looks optically transparent, but after the condensation several white “holes” appear randomly distributed. “Holes” are not present immediately after the deposition (Figure S4a), thus the combination of mechanical stresses, due to the vapor shear stress and the condensate flow, and chemical harsh environment, causes the degradation of the hydrophobic coating. Once the coating is damaged, the wettability of both aluminum and aluminum hydroxide at the bottom becomes important, increasing the global wettability of the surface. As a result, the contact angles measured after the condensation test are lower than those measured before.

#### 3.1.2. Surface Chemistry Investigation

**Figure 3:**
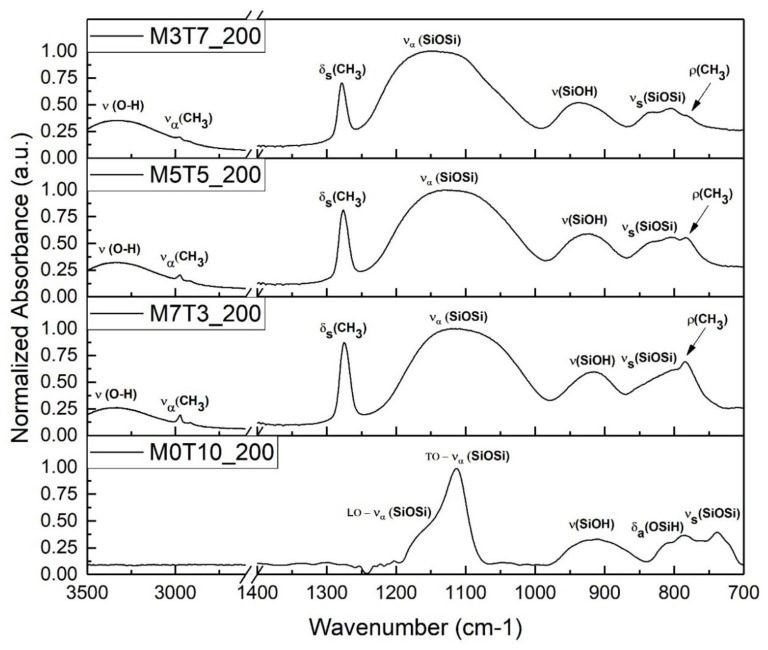
Fourier-Transform Infrared (FTIR) spectra of M7T3 coatings changing the precursors’ concentration, compared with a silica coating.

The presence of the methyl group of the MTES precursor in the different coatings was verified by the FTIR technique. Analysis of the IR spectra can be used as an indicator of possible variation in the molecular structure before and after the condensation tests as well. In Figure 3, comparison of spectra collected from an organic modified silica film and a silica film (M0T10_200) is reported. The same spectra show also the evolution of methyl groups due to different precursors’ molar ratios.

As already known by previous work [23], aluminum has not vibrations in the chosen range. The most intense peak is related to vibration of the silica network and it is usually split by long-range coupling Coulomb interactions in a transverse optical mode (TO) and in a longitudinal optical mode (LO) [24]. The peak is centered for the M0T10_200 sample at 1110 cm^−1^, caused by the asymmetric stretching of the Si–O–Si bond, due to vibration mode, while the LO vibration mode is centered around 1160 cm^−1^ and it is detectable as a shoulder at the high-frequency end of the 1110 cm^−1^ peak [25,26]. When methyl group is introduced in the silica film, IR spectra exhibit some typical vibrations coming from the organic groups, especially in the range 3000–2800 cm^−1^ and 1300–1200 cm^−1^. In particular, the clearest peak is the one centered at 1270 cm^−1^, not present in the pure silica film spectra, and coming from the vibration of the methyl attached to Si [20,25,27,28,29]. Other bands related to Si-CH_3_ vibrations are less visible because they are partially overlapped and characterized by lower intensity, e.g., around 2950 cm^−1^, the –CH_3_ vibrations are overlapped by the board peak in the 3500–3000 cm^−1^ range, related to the O–H stretching of the physisorbed water and to the unreacted Si–OH stretching; around 780 cm^−1^ the C–H vibration is overlapped by the broadband of the Si–O–Si bond vibration [20,30]. In the hybrid films, the formation of a silica’s broad peak makes harder to distinguish the two vibration modes. Hydrolyzed but not condensed precursor was found in the analyzed baking conditions (200 °C), resulting in the formation of a band at 915 cm^−1^ related to Si-OH or SiO^-^ vibrations [20].

As expected [20,27], increasing the amount of MTES causes an increase of the organic groups in the deposited film, and the normalized intensity of the bands corresponding to the organic vibrations rise up. It is worth noting that this trend is evident in the overlapped bands as well: at 780 cm^−1^ the methyl’s vibration starts as shoulder of the Si–O broadband in M3T7_200 and gradually becomes the predominant peak in M7T3_200. Similarly, the –CH_3_ stretching peak in the O–H broad band centered at 2950 cm^−1^ in M3T7_200, becomes a clear peak in M7T3_200. The number of organic groups seems to not affect directly the condensation of the silica network, due to the constant intensity of the peak related to Si–OH vibration in all normalized spectra.

**Figure 4:**
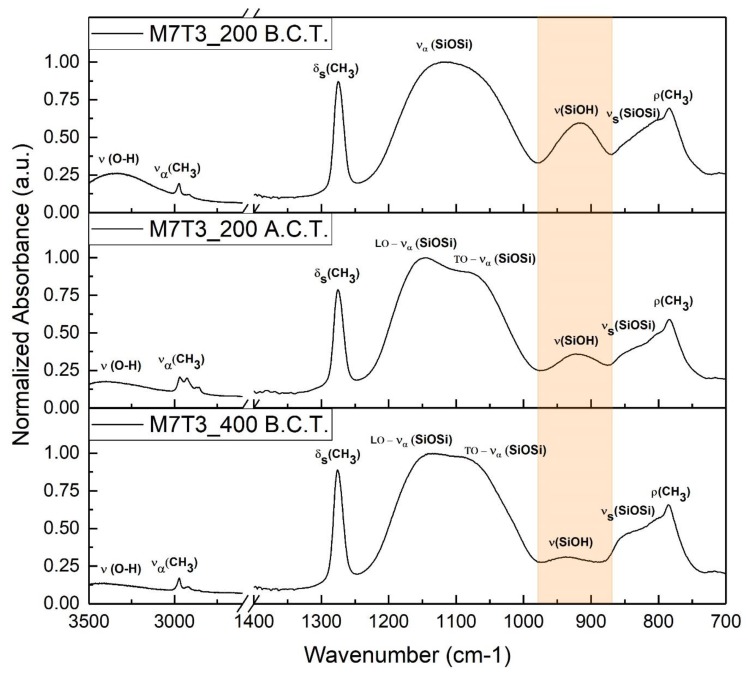
FTIR spectra of M7T3_200 (before and after condensation test, B.C.T. and A.C.T., respectively) and M7T3_400 coatings.

In order to study the thermal evolution of the coating and the possible effect in the chemical nature of steam’s exposure, normalized IR spectra of M7T3 films increasing the thermal annealing and before and after the condensation test are reported in Figure 4.

Methyl groups in the coatings are unaffected by the baking temperature up to 400 °C, as studied by other authors [20], in fact, the methyl’s related bands have similar normalized intensity in the two spectra collected before the condensation test. Vice versa, increasing the annealing temperature, the condensation of the film and the physisorbed water removal become higher. These effects are evident in the dramatic decrease of both O–H broadband in the 3500–3000 cm^−1^ range and Si–OH peak with a baking temperature of 400 °C; while peaks related to the silica network become more defined. In fact, at higher temperatures, the double peak related to TO and LO vibration modes of Si–O–Si linkage become detectable and the shoulder at 850 cm^−1^ related to Si–O–Si vibration increases in intensity.

The M7T3_200 coating is evaluated also after the condensation test to address the possibility of a physiochemical interaction with the steam. The most evident difference is the signal drop coming from the unreacted silanol groups centered around 920–915 cm^−1^ and highlighted with an orange box. As a result, a higher condensation of the silica network occurs during water condensation tests, in accordance with the results obtained by H. Imai et al. [31] for TEOS coatings.

#### 3.1.3. Thickness Measurement

The good transparency of the sol–gel layers allows us to evaluate the thickness of the coatings by ellipsometry. The results of the data fitting are reported in Table 2:

All samples show similar thickness in the 200–250 nm range. A decrease is observed after the condensation tests due to the film’s shrinkage and degradation, with a final mean value around 100–150 nm. The coating becomes gradually opaquer and less describable by the Cauchy Transparent Dispersion model, as consequence the Mean Square Error (M.S.E.) after the condensation test becomes higher with respect to the one before the condensation test.

### 3.2. Heat Exchange Tests

#### 3.2.1. DWC Measurements

**Figure 5:**
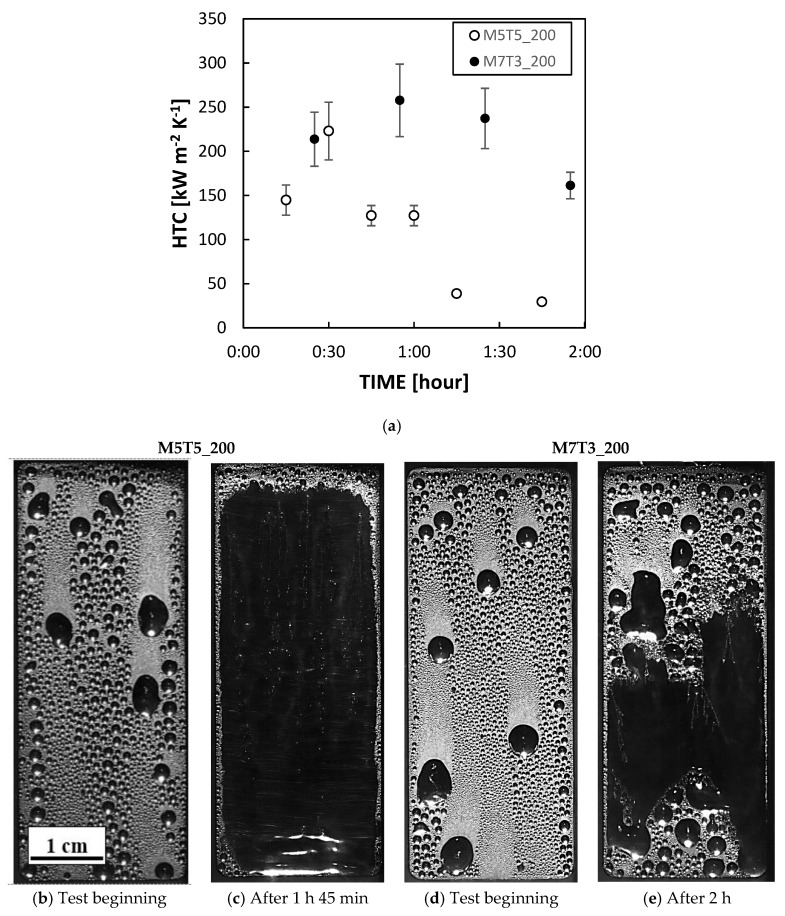
(**a**) Heat transfer coefficient (HTC) of M5T5_200 and M7T3_200 samples. (**b**–**e**) Images recorded at the beginning and at the end of the condensation test for M5T5_200 and M7T3_200, respectively. All data were recorded at constant saturation temperature (105 °C), constant vapor velocity (2.6 m s^−1^) and constant heat flux (400 kW m^−2^).

**Figure 6:**
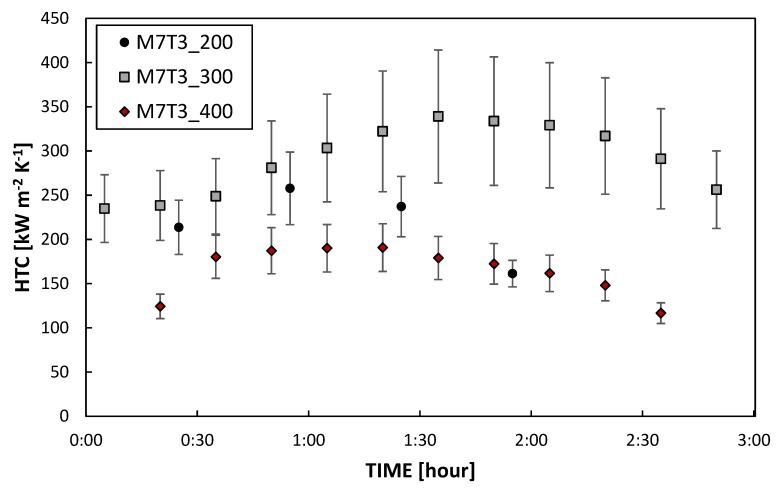
HTC of M7T3_200, M7T3_300 and M7T3_400 coatings. All data were recorded at constant saturation temperature (105 °C), constant vapor velocity (2.6 m s^−1^) and constant heat flux (400 kW m^−2^).

As described in the previous section only the coatings with the higher content of MTES (i.e., M5T5 and M7T3) were tested in the condensation chamber. All the tested coatings promoted DWC on aluminum substrates, showing a stunning HTC augmentation of around one order magnitude, in agreement with [21]. However, the duration of the hydrophobic layers deposited on the aluminum substrate was limited over time and hence the evolution of the process, from the start to the end of the condensation test, was analyzed. All the samples were tested at *T_SAT_* = 105 °C, *v_VAP_* = 2.6 m s^−1^ and heat flux equal to 400 kW m^−2^. All data reported are measured with the two thermocouples placed in the middle of the sample in order to avoid boundary effects. All coatings display the same DWC evolution (fully illustrated in Section 3.2.2 for sample M7T3_300): first pure DWC, then DWC with elongated drops and, in the end, the bottom of the surface there is a gradual transition to FWC with the flooding of the surface. The effect of the MTES concentration is presented in Figure 5, where the HTCs during steam condensation measured on M5T5_200 and M7T3_200 (Figure 5a) and the images at the beginning and at the end of the test (Figure 5b–e) are shown. The M5T5_200 coating showed a weak sturdiness in such an environment: after just 30 min the HTC started to decrease (Figure 5a) and the surface was completely flooded after two hours (Figure 5c). Instead, M7T3_200 coating showed an HTC peak at around 1 h and only around 30% of the surface was flooded after 2 h (Figure 5e). Videos of the initial stage (Video S7) and the final stage (Video S8) are reported in the Supporting Materials for M7T3_200. The different lifetime of samples at the same annealing conditions can be related to the amount of MTES. As already described by Innocenzi et al. [20], increasing the number of organic groups the porosity of the layer decrease, with the formation of smaller pores and making them more spherical and hydrophobic. On the other hand, the same authors correlated the less amount of OH groups that can condensate with the higher thermal stability of M7T3_200 with respect to M5T5_200. When comparing the results obtained on the M7T3_200 sample and presented in this paper with those reported in our previous study on samples with the same composition [7], it can be seen that the lifetime here is shorter and it can be ascribed to the higher heat flux tested in the present study. Experimentally, it was observed that the heat flux has a great impact on the degradation, resulting in shorter lifetime at higher heat flux. From the point of view of durability, the most promising sample is therefore the M7T3, which was fully investigated in Figure 6, changing the baking temperature.

With regard to HTC, its value is strongly influenced by the coating thermal resistance [32,33], defined as the ratio of coating thickness to coating thermal conductivity. Commonly, the thermal conductivity of these coatings is set equal to 0.2 W m^−1^ K^−1^. As a result, the value of HTC is strongly dependent on the coating thickness. It is interesting to notice that the thickness value changes significantly if it is measured before or after condensation tests (see Table 2) and this may explain the initial HTC increase with time.

All the layers displayed similar thickness (See Section 3.1.3) and therefore the thermal resistance associated with it should be very similar. The sturdiness of the coatings, instead, seems to be different under the same operating conditions. Increasing the annealing temperature seems to improve the sturdiness of the coating, increasing the coating lifetime from less than 2 h (M7T3_200) up to around 3 h (M7T3_300). The M7T3_400 lasts around 2 h and half, leaving the 300 °C baking temperature as optimum temperature for DWC promotion. As discussed in Section 3.1.2, the baking temperature changes the characteristics of the sol–gel coating, changing the degree of ramification of the network. As already described by other authors [20] different connectivity in the network affect the mechanical and the physicochemical proprieties, especially referred to as porosity and flexibility of the chain. In the next section, the DWC evolution of M7T3_300 will be fully analyzed since it gave the best results in terms of durability.

#### 3.2.2. DWC Evolution

**Figure 7:**
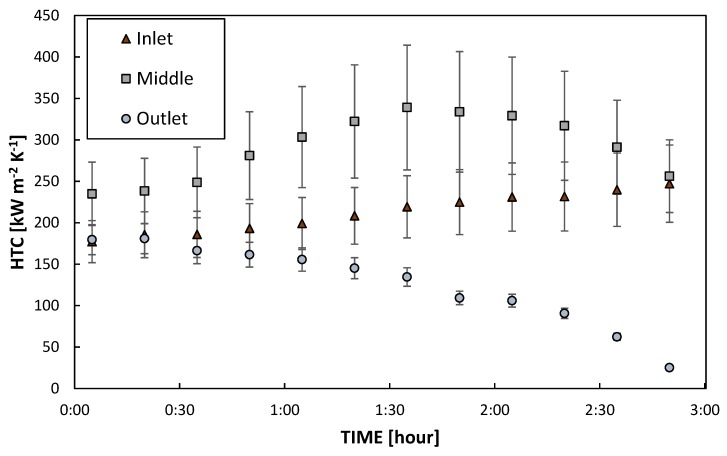
Time evolution of the HTC during dropwise condensation (DWC) on M7T3_300 sample in different areas.

In Figure 7, the HTC measured at fixed operating conditions (see Section 3.2.1) on M7T3_300 is reported for three positions called Inlet, Middle and Outlet (see Figure S1 in the Supporting Materials). Although all the HTCs remained constant for about 30 min, the coating degradation is not homogenous along the aluminum substrate. The HTC measured at the inlet and at the middle increases over time, in particular, the HTC measured at the middle reaches a maximum value after 1 h and 30 min from the beginning of the test and, then, starts to decrease. The HTC measured at the outlet, instead, decreases for all the 3 h of the experimental campaign.

Since the degradation may differ among different areas of the sample, the HTC defined in different areas may consequently vary. This may be an explanation of why the HTC values presented here slightly differ from the values reported in [7], where it was defined in a different area.

**Figure 8:**
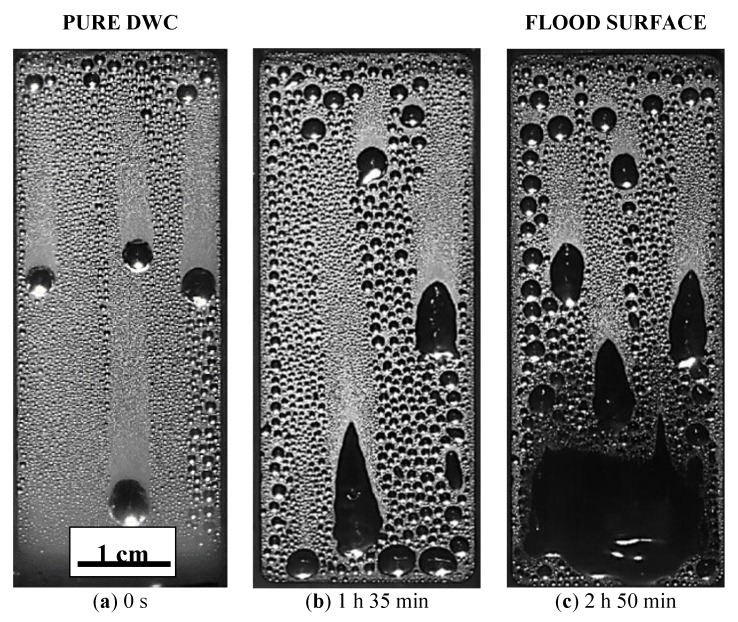
Images recorded during condensation tests on M7T3_300 at a different time steps: (**a**) at the beginning of the experiment, (**b**) after 1h and 30min, (**c**) after 2h and 50min.

Similar degradation trends with time have already been found for other coatings [34], meaning that coatings change over time due to the harsh environment of the condensation experiments. In particular, such high temperatures (around 100 °C) of saturated steam induce shrinkage of the sol–gel treatments, as reported in [31]. The HTC variation over time can be explained by the modification of one of the two components of the coating thermal resistance: the coating conductivity and the coating thickness. Since the coating thermal conductivity should remain constant, the coating thickness can be the main responsible for the HTCs evolution. The coating shrinkage may reduce the thermal resistance and, thus, increase the HTC [7]. On the other hand, looking at Figure S3, the thermal stress can induce the gradual deterioration of the hydrophobic layer, mostly at the outlet. The “holes” in the coating leaves the aluminum substrate exposed, which has a higher wettability, where the condensate can be stuck on the surface. In this case, thermal resistance is added to the process, the resistance of the condensate, and thus the HTC starts to decrease. The HTC evolution can be also compared with the evolution of the droplets at different stages of the condensation test, as presented in Figure 8 (the related videos are reported in the Supporting Materials). The droplets’ shape changes from a quasi-spherical shape maintained all along the sample (Figure 8a and Video S9) to an elongated one after about one hour and a half (Figure 8b and Video S10). At the end of the test, the bottom of the surface is wetted by a continuous layer of condensate (Figure 8c and Video S11). Since the vapor flow should be pretty constant along the sample and a chemical attack should be quite homogenous, the cause of the different degradation with respect to the position along the vapor flow could be attributed to the condensate shedding. Since at the outlet the condensate mass flow rate is the highest, the degradation is faster in this position.

The flooding of the surface is also an indicator that the surface wettability is changed. Contact angle analysis, reported in Figure 1 and Figure 2 shows that both advancing and receding contact angles decrease from their initial values, but looking at the local values of the angles measured at the inlet and at the outlet of the sample, the advancing contact angle was around 60° in both the cases, whereas the receding contact angles was around 30° at the inlet and 15° at the outlet. Since at the inlet position DWC is still occurring (see Figure 8) while at the outlet the surface is flooded, the receding contact angle plays here a key role in the condensation mode transition. It is interesting to relate the changed wettability to a modification of the surface morphology as reported by SEM images taken after the condensation test at the inlet and at the outlet position (see Figure S4). In particular, at the bottom of the sample where the “holes” density is higher, the receding contact angle is the lowest and the surface resulted to be flooded by the condensate. This is in agreement with several studies reported in literature [34,35] that demonstrated how the surface wettability is mostly influenced by the receding contact angle. Due to small dimension of the “holes”, in loco analysis was not possible to be done, but correlating the results with a bare aluminum sample tested in the same experimental conditions, it is reasonable to suppose the formation of aluminum hydroxide at the bottom of the “holes” or the exposure of bare Aluminum due to coating’s removal (See Figures S5 and S6). Both metal and metal hydroxide are characterized by high water’s wettability. Furthermore, also the droplet departing radius is affected by the decrease of the dynamic contact angles. The droplet departing radius measured in Figure 8 at 0 s is equal to 1.2 mm ± 0.1 mm, it increases up to 1.4 mm ± 0.1 mm after 2 h and 50 min, consistently with the decrease of both θ_a_ and θ_r_ angles and the increase of the contact angles hysteresis [32,33].

## 4. Conclusions

Hybrid organic-inorganic sol–gel silica coatings have been studied as promising solutions for DWC promotion. In particular, the mix of methyl triethoxy silane (MTES) and tetraethyl orthosilicate (TEOS), labeled MxTy, has been investigated with different MTES/TEOS molar ratios and annealing temperatures. Six different combinations have been characterized in terms of wettability, surface composition and morphology before and after condensation tests. Although the different coatings initially presented similar wetting characteristics and chemistry, they displayed different durability during the condensation test campaign. All the tested coatings promoted DWC, with a maximum heat transfer coefficient of 300 kW m^−2^ K^−1^ which is one order magnitude higher compared to FWC in the same operating conditions. However, the environmental conditions during the condensation test (high temperatures, vapor shear stress) led to a degradation of the coatings. The coating composed of 70% of MTES and 30% of TEOS annealed at 300 °C showed the best performance in terms of durability at fixed steam parameters, lasting for 3 h. A detailed analysis has been proposed for understanding the sol–gel coatings degradation during the condensation tests. SEM images show the presence of holes in the coatings, which are not homogeneously distributed over the substrate, but seriously affect the surface wettability, thus explaining why the HTC varies with time and it is different in different areas of the sample.

## Figures and Tables

**Table 1:** List of the studied samples.

Name	MTES Molar Percentage	TEOS Molar Percentage	Baking Temperature
M0T10_200	0%	100%	200 °C
M3T7_200	30%	70%	200 °C
M5T5_200	50%	50%	200 °C
M7T3_200	70%	30%	200 °C
M7T3_300	70%	30%	300 °C
M7T3_400	70%	30%	400 °C

**Table 2:** Measured thickness by ellipsometric technique before and after the condensation test.

	Before Condensation Test			After Condensation Test		
ID	n (640 nm)	Thickness	M.S.E.	n (640 nm)	Thickness	M.S.E.
M0T10_200	1.4372	173.2 nm	1.96	/	/	/
M3T7_200	1.4226	196.4 nm	3.38	/	/	/
M5T5_200	1.4445	226.0 nm	1.45	1.4088	154.78 nm	5.10
M7T3_200	1.4252	252.60 nm	2.74	1.3832	115.65 nm	11.11
M7T3_300	1.4216	229.36 nm	1.71	1.3920	125.32 nm	13.12
M7T3_400	1.3945	199.27 nm	2.90	1.3896	120.12 nm	12.18

## Supplementary Materials

The following are available online at https://www.mdpi.com/1996-1944/13/4/878/s1, Sketch of the experimental rig (Figure S1). Contact angles of the silica film with different MTES/TEOS ratios and different thermal annealing before and after condensation tests (Table S2). Optical images of aluminum substrates coated with M7T3_200 before and after the condensation (Figure S3). SEM images and XRD pattern of samples (Figures S4–S6). Videos of dropwise condensation (S7–S11).

## Nomenclature

*HTC*	Heat transfer coefficient (between steam and metallic substrate), kW m^−2^ K^−1^
*k*	coverage factor, -
*q*	Heat flux, kW m^−2^
*T*	Temperature, K
*z*	Orthogonal axis of the sample, m
*Greek symbols*	
*ΔT*	Temperature difference, K
λ	Thermal conductivity, W m^−1^ K^−1^
*θ*	Contact angle, °
*Subscripts*	
a	Advancing
r	Receding
sat	Saturation
wall	Surface
