# Supplementary material for: Optimization of Hybrid Sol-Gel Coating for Dropwise Condensation of Pure Steam
Source: Materials (Basel). 2020 Feb 15;13(4):878. doi: 10.3390/ma13040878 (PMC7078621; doi:10.3390/ma13040878)
Supplement: Supplementary file 1 [file materials-13-00878-s001.zip › SM/materials-705053-supporting materials_final.docx]

**Supplementary Materials**

Optimization of Hybrid Sol-Gel Coating for Dropwise Condensation of Pure Steam

Riccardo Parin^a^, Michele Rigon^a^, Stefano Bortolin^a^, Alessandro Martucci*^a^ and Davide Del Col^a^

^a^ Industrial Engineering Department, University of Padova, Via Venezia 1, 35131 Padova, Italy


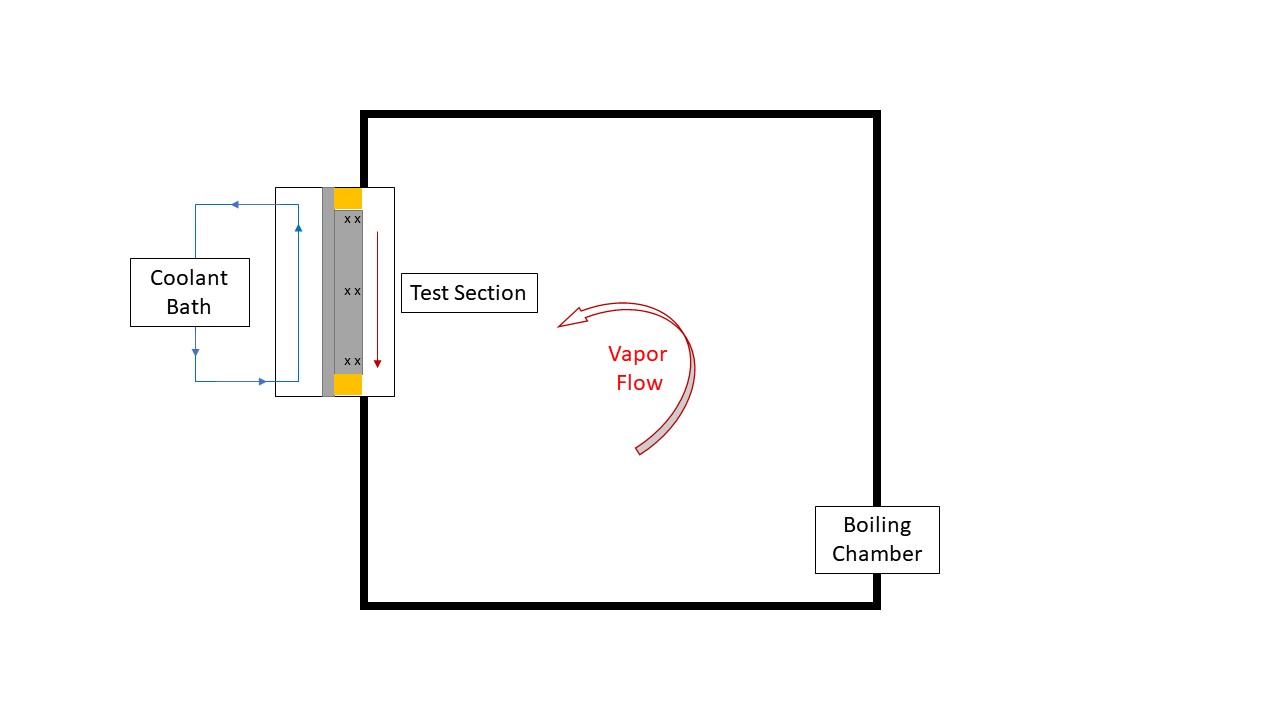


Middle

Inlet

Outlet

**Figure S1.** Sketch of the experimental rig

**Table S2.** Contact angles of the silica film with different MTES/TEOS ratios and different thermal annealing before and after condensation tests.

|  | **Before Tests** | | **After Tests** | |
| --- | --- | --- | --- | --- |
| **Samples** | **Θa** | **Θr** | **θa** | **θr** |
| Bare Aluminum | 70°±1° | <10°* | 78°±5° | <10°* |
| M0T10 | 46°±4° | 16°±1° | \ | \ |
| M3T7_200 | 84°±1° | 60°±2° | \ | \ |
| M5T5_200 | 84°±3° | 61°±4° | 53°±5° | <10°* |
| M7T3_200 | 81°±3° | 65°±3° | 65°±7° | 27°±5° |
| M7T3_300 | 81°±3° | 67°±3° | 63°±3° | 22°±6° |
| M7T3_400 | 81°±2° | 65°±2° | 51°±7° | 16°±3° |

* Flattened drops cannot be fitted exactly with sessile drop method. Contact angles in this case are indicated with “< 10°”.

| *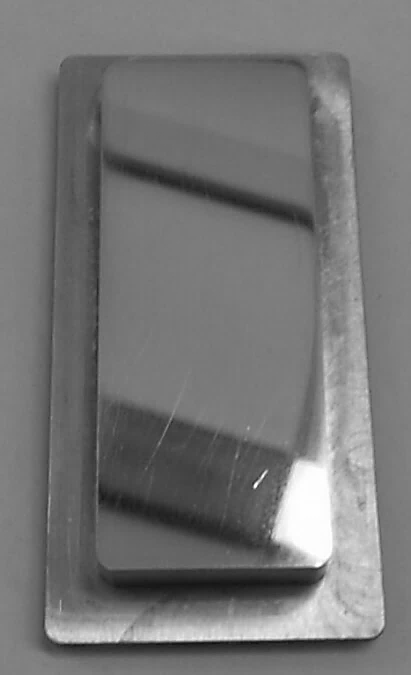* | *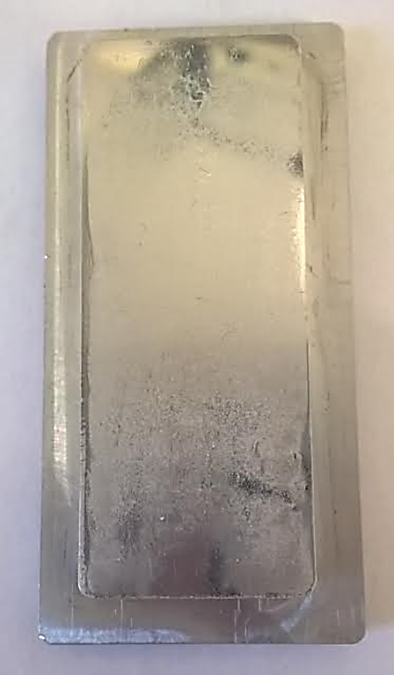* |
| --- | --- |
| *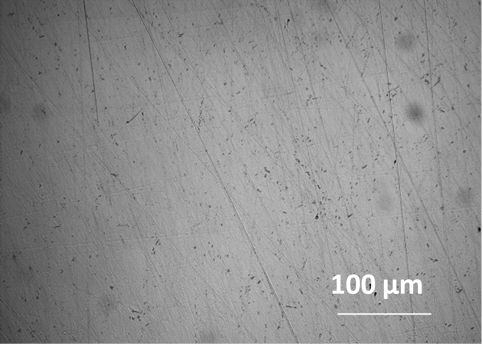* | *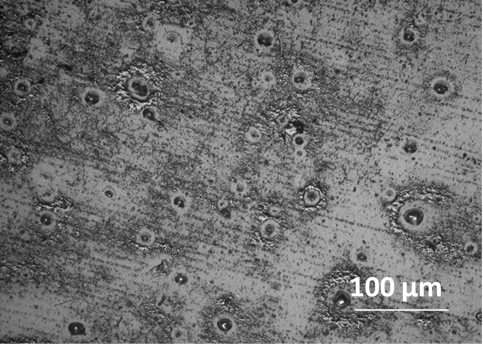* |
| **Before Condensation Test** | **After Condensation Test** |

**Figure S3.** (top) Images of the 50mmx20mm aluminum substrates coated with M7T3_200 and (bottom) optical microscope magnification of the indicated area, before and after the condensation test.

| a) | 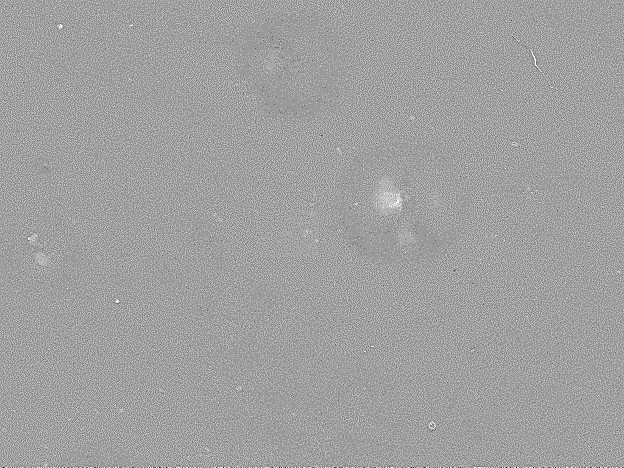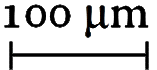 | b) | 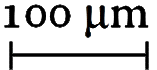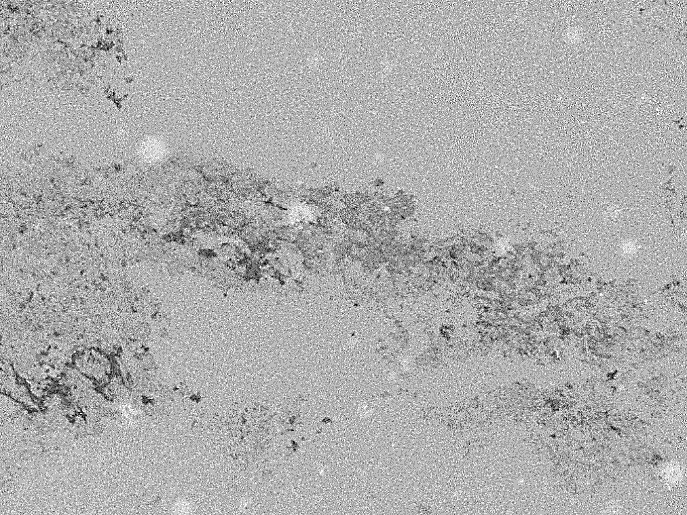 |
| --- | --- | --- | --- |
| c) | 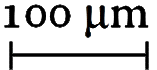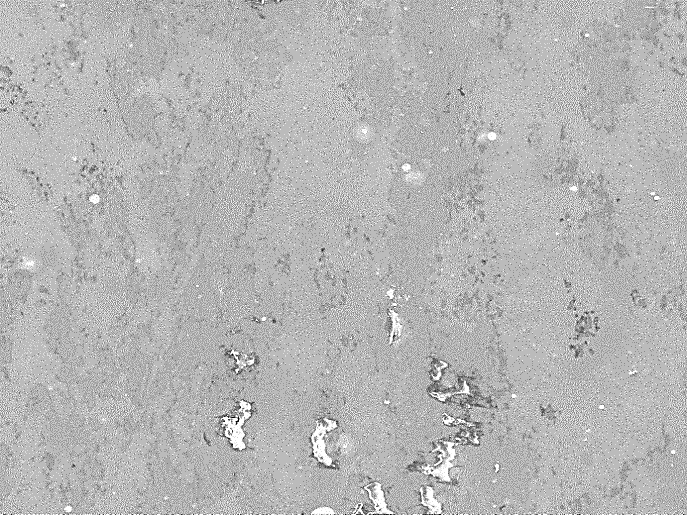 | d) | 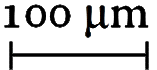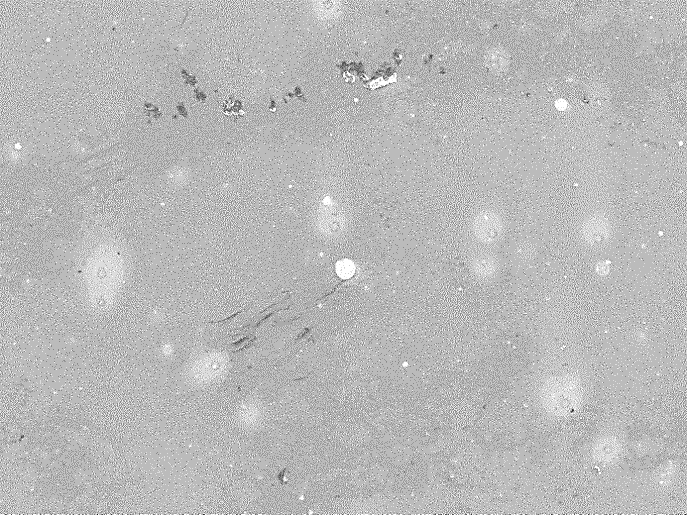 |

**Figure S4.** SEM images of sample M7T3_200 before (a) and after the condensation test in inlet position (b), in middle position (c), and in outlet position (d), according with section 3.2.2 nomenclature.

**Figure S5.** XRD pattern of bare aluminum sample after the condensation test showing the formation of aluminum hydroxide (boehmite, ICDD Card 76-1871). The aluminum diffraction peaks (ICDD Card 04-0787) are also evident.

| *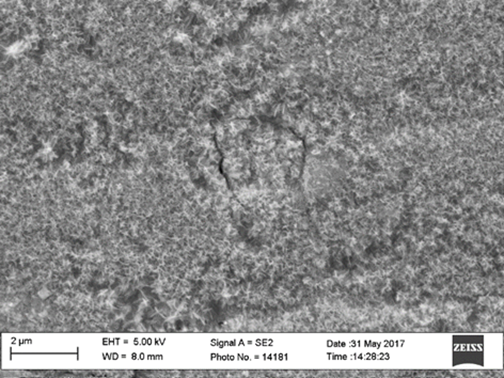*a)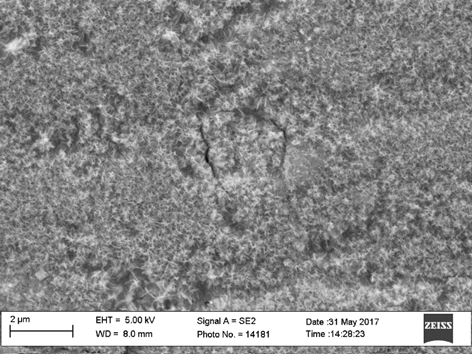 | 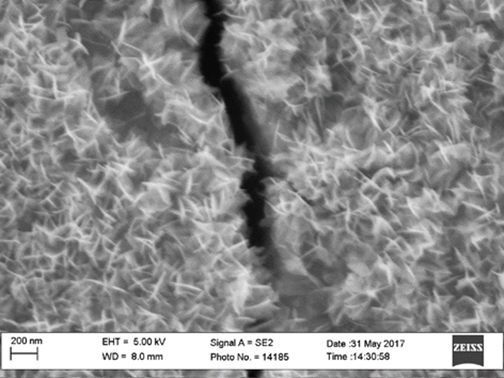b)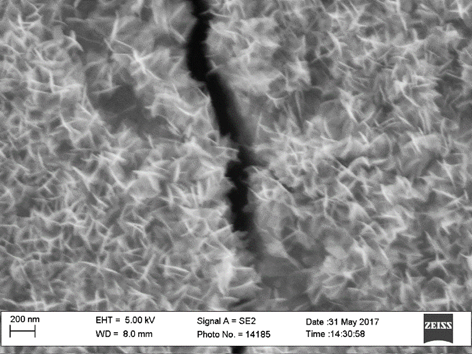 |
| --- | --- |

**Figure S6.** SEM images of bare aluminum sample after the condensation test showing the formation of typical aluminum hydroxide (boehmite) microstructures, more evident at higher magnification (figure b).

| 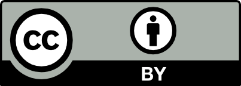 | © 2020 by the authors. Submitted for possible open access publication under the terms and conditions of the Creative Commons Attribution (CC BY) license (http://creativecommons.org/licenses/by/4.0/). |
| --- | --- |
